# The Diagnostic Utility of Reticulocyte Hemoglobin in Iron Deficiency Anemia and Early Response Following Intravenous Iron Therapy

**DOI:** 10.7759/cureus.113999

**Published:** 2026-08-05

**Authors:** Mahadev Meena, Tarini Prasad Dandasena, Garima Goel, Asha Meena, Sachin Bansal, Rajnish Joshi

**Affiliations:** 1 Internal Medicine, All India Institute of Medical Sciences Bhopal, Bhopal, IND; 2 Gastroenterology, All India Institute of Medical Sciences Gorakhpur, Gorakhpur, IND; 3 Pathology, All India Institute of Medical Sciences Bhopal, Bhopal, IND; 4 Pathology, Sawai Man Singh Medical College, Jaipur, IND; 5 Clinical Hematology, LN Medical College and Research Centre, Bhopal, IND

**Keywords:** intravenous iron therapy, iron deficiency anemia, iron profile, multichannel hematological autoanalyzer, reticulocyte hemoglobin

## Abstract

Background and objectives

Iron deficiency anemia (IDA) remains a major global health concern, with diagnostic challenges arising from the limitations of traditional biochemical markers. This study aimed to evaluate the diagnostic utility and reliability of reticulocyte hemoglobin (RET-Hb) in identifying IDA and assessing the early functional response, defined as any change in RET-Hb within 48 hours of intravenous iron infusion, as a marker of early marrow iron availability. Specifically, the study sought to assess the percentage change in RET-Hb from baseline following iron infusion. Furthermore, the correlations between RET-Hb and traditional iron indices were evaluated.

Methods

A single-center combined observational design, including a case-control and longitudinal (within-subject) study, was conducted over 12 months in the Department of General Medicine, All India Institute of Medical Sciences (AIIMS) Bhopal. The study included 200 participants, with 100 patients diagnosed with IDA and 100 healthy controls. RET-Hb and other hematological and biochemical parameters were measured at baseline and at 48 hours after intravenous iron therapy in patients receiving intravenous iron as part of standard care. Statistical analyses included the Shapiro-Wilk test, Spearman's rank correlation, paired t-tests, and receiver operating characteristic (ROC) curve analysis. The diagnostic performance of RET-Hb was also compared with that of serum ferritin, transferrin saturation (TSAT), serum iron, mean corpuscular volume (MCV), and mean corpuscular hemoglobin (MCH).

Results

RET-Hb levels were remarkably lower in IDA patients compared to controls (26.68 ± 4.04 vs. 32.68 ± 1.94 pg; p < 0.001). RET-Hb demonstrated strong positive correlations with serum iron (r = 0.631), serum ferritin (r = 0.672), and TSAT (r = 0.619), and a negative correlation with total iron-binding capacity (TIBC) (r = -0.571). ROC analysis identified a RET-Hb cutoff of 29.65 pg, which predicted IDA with 73% sensitivity and 100% specificity (area under the curve (AUC) = 0.895). RET-Hb increased within 48 hours of treatment, indicating its utility as an early marker of improvement in functional iron availability, preceding measurable changes in hemoglobin (Hb). Overall, 80% of cases demonstrated measurable improvement. The most common category of change was a 1-2 pg increase, observed in 35% of patients, while 14% experienced a rise of more than 5 pg.

Conclusions

RET-Hb demonstrated good diagnostic performance for identifying IDA in this study and showed an early increase following intravenous iron therapy, suggesting an improvement in functional iron availability. However, because the study compared patients with confirmed IDA with healthy controls, the observed diagnostic performance should be interpreted cautiously and requires validation in more diverse clinical populations.

## Introduction

Iron deficiency remains the predominant contributor to anemia worldwide. While nutritional iron deficiency predominates in low-resource settings, chronic blood loss represents the leading cause globally. Successful management of iron deficiency anemia (IDA) depends on accurate diagnosis, identifying the underlying cause of iron depletion, and providing appropriate iron supplementation for an adequate duration. When treating IDA, clinicians must be vigilant about possible genetic causes, as these can affect the absorption, transport, or utilization of iron. Commonly used biochemical markers for diagnosing IDA include ferritin, total iron-binding capacity (TIBC), serum iron, transferrin saturation (TSAT), and mean corpuscular volume (MCV).

Ferritin (reflecting iron storage in the body) is frequently considered a key marker for detecting iron deficiency. However, its reliability diminishes in the presence of inflammation, as inflammatory states can falsely elevate ferritin levels (acute phase reactant), masking underlying iron deficiency. Serum iron is mainly influenced by recent dietary intake, while TIBC decreases with various inflammatory conditions. As serum iron and TIBC are influenced by various physiological and pathological conditions, TSAT, which is derived from these parameters, can vary accordingly [1]. In conditions such as malnutrition or chronic illness, hepatic production of transferrin may decline. When serum iron stays relatively stable, this fall in transferrin can make TSAT look higher than it really is. A low TSAT (< 16-20%) measured after five to nine hours of fasting is a reliable indicator of iron deficiency; however, Nguyen et al. reported no significant reduction in variability in their epidemiological study. Although some studies indicate statistically significant diurnal variations in body iron levels, the findings are not consistent [2,3].

Normally, reticulocytes constitute approximately 1% (0.5%-2.5%) of circulating erythrocytes. Measuring the reticulocyte count provides insights into bone marrow activity and its response to anemia, reflecting the rate of erythropoiesis. Reticulocyte hemoglobin (RET-Hb) has emerged as a useful adjunctive biomarker for assessing iron availability for erythropoiesis and, when interpreted alongside conventional iron indices, may support the early identification of iron deficiency while reducing diagnostic misclassification. This is because hemoglobin (Hb) synthesis is affected first in reticulocytes. Reticulocytes, with a short lifespan of approximately two to three days, provide an immediate representation of iron utilization during erythropoiesis. RET-Hb measurements are less influenced by most physiological changes, although conditions such as macrocytosis or thalassemia can still affect the results [4,5]. Importantly, RET-Hb is now easily accessible as part of a complete blood count (CBC) performed on modern multichannel hematology analyzers, making it a practical and efficient tool for routine clinical assessments. It represents one of the promising parameters that can complement existing diagnostic approaches.

Furthermore, changes in RET-Hb may occur earlier than changes in conventional reticulocyte counts or Hb levels, suggesting that it could be a useful adjunctive marker of early improvement in functional iron availability following iron therapy. RET-Hb is a direct measure of the Hb content in newly formed reticulocytes, reflecting iron availability for erythropoiesis. It may serve as a dynamic marker of treatment response and changes in functional iron availability because reticulocytes have a short lifespan before maturing into erythrocytes. RET-Hb may play a vital role in distinguishing early responders from non-responders to iron therapy.

This may help clinicians explore underlying causes of iron deficiency, such as malabsorption in patients receiving oral iron, iron-refractory iron deficiency anemia (IRIDA), or ongoing occult blood loss. In the early stages of inflammation, iron is redistributed into macrophages rather than being available for red blood cell production, even though total body iron stores remain largely unchanged. Our study excluded patients with inflammatory conditions. A few studies have also highlighted the value of RET-Hb in optimizing iron therapy, particularly in individuals with chronic kidney disease undergoing treatment with erythropoiesis-stimulating agents. RET-Hb may serve as a useful marker for tailoring iron therapy in the future, given its ability to reflect current iron availability.

The present study was designed to evaluate the diagnostic utility of RET-Hb for identifying IDA and to assess the early change in RET-Hb within 48 hours of intravenous iron infusion as a marker of early marrow iron availability. An additional objective of the study was to assess the correlation between RET-Hb levels and conventional iron indices.

## Materials and methods

In this study, we utilized a combined observational design including case-control and longitudinal study (within-subject). It was conducted in the Department of General Medicine, All India Institute of Medical Sciences (AIIMS), Bhopal, over a period of 12 months, from March 2022 to March 2023, to assess the diagnostic utility of RET-Hb. After ethical approval, a total of 200 participants were included in this study. The sample size was estimated using a precision-based approach for a case-control diagnostic accuracy study (see Appendices). Of these, 100 participants were diagnosed with IDA based on clinical and laboratory parameters. Patients with IDA were classified as cases. Another 100 age- and sex-matched participants without evidence of anemia and with a normal hemogram were considered the controls. We compared both groups to evaluate differences in hematological parameters, iron profiles, and RET-Hb. We also evaluated the utility of RET-Hb as an adjunctive parameter for identifying IDA.

Patients diagnosed with IDA received a single 1000 mg dose of intravenous (IV) ferric carboxymaltose, administered as an intravenous infusion in accordance with the institutional protocol under routine clinical monitoring. Patients were observed for immediate infusion-related adverse events during and after administration. Hematological parameters, including Hb, MCV, MCH, mean corpuscular hemoglobin concentration (MCHC), red cell distribution width coefficient of variation (RDW-CV), and RET-Hb, were measured at baseline and 48 hours post-treatment to assess the early functional response. Early functional response was operationally defined as any change in RET-Hb from baseline at 48 hours after intravenous iron infusion.

Patients with dimorphic anemia, known hematological disorders (such as hemoglobinopathies), hyper-segmented neutrophils on peripheral blood film, or macrocytic anemia (MCV > 100 fL), as well as those with elevated C-reactive protein (CRP) levels (> 3 mg/L), those undergoing chemotherapy or radiotherapy, pregnant individuals, or those who had received recent iron treatment or were currently receiving oral iron supplementation were excluded. Anemia was defined according to Hb thresholds of less than 12.0 g/dL for women and less than 13.0 g/dL for men. IDA was diagnosed using a predefined composite reference standard. Patients were required to have anemia according to WHO criteria together with biochemical evidence of iron deficiency, defined by serum ferritin below the laboratory reference range and/or TSAT below the predefined threshold, in conjunction with supportive iron studies (serum iron and TIBC), after exclusion of alternative causes of anemia. Clinical assessment and laboratory findings were considered together before assigning the diagnosis.

The ferritin threshold was selected in accordance with contemporary recommendations that serum ferritin concentrations below approximately 30 ng/mL are highly suggestive of iron deficiency in the absence of inflammation. A threshold of < 25 ng/mL was chosen as the laboratory diagnostic cut-off used at our institution to improve specificity while maintaining sensitivity for iron deficiency in individuals with normal CRP levels. A serum ferritin level of < 15 µg/L is indicative of absent iron stores, while serum ferritin levels below 30 µg/L are generally indicative of low body iron stores. Therefore, the lower limit of normal for most laboratories lies within the range of 15-30 µg/L (as per the British Society of Gastroenterology guidelines for the management of iron deficiency anemia in adults, 2021).

Blood samples for complete blood count and RET-Hb measurement were collected in ethylenediaminetetraacetic acid dipotassium salt (EDTA-2K)-containing vacutainers, and an automated hematological analyzer (Sysmex, XN-1000, Sysmex Corporation, Kobe, Japan) was used for analysis. For CRP, serum iron, TIBC, and serum ferritin assessments, blood samples were collected in regular vacutainers containing gel and clot activators and analyzed using a Coulter AU 680e automated chemical analysis device (Beckman Coulter, Brea, CA). Baseline blood samples were collected before the administration of intravenous iron. Follow-up samples were obtained approximately 48 hours after completion of the infusion using the same laboratory procedures.

Data were collected using a pre-designed structured proforma and entered into Microsoft Excel. Statistical analyses were performed using IBM SPSS Statistics version 22 (IBM Corp., Armonk, NY) for Windows. Continuous variables were assessed for normality using the Shapiro-Wilk test and visual examination of histograms. Normally distributed variables were expressed as mean ± standard deviation (SD), whereas non-normally distributed variables were summarized as the median and interquartile range (IQR). Categorical variables were presented as frequencies and percentages. Comparisons between cases and controls were performed using the independent-samples Student’s t-test or an appropriate non-parametric test, depending on data distribution. Changes in laboratory parameters before and after treatment within the case group were assessed using the paired t-test or the corresponding non-parametric test. Spearman’s rank correlation coefficient was used to assess the association between RET-Hb and other laboratory parameters, including serum ferritin, TSAT, serum iron, TIBC, MCV, and MCH, because several parameters were not normally distributed.

The distribution of continuous variables was assessed using the Shapiro-Wilk test and visual inspection of histograms (see Appendix). Because RET-Hb and most conventional hematological and iron indices were not normally distributed, associations between RET-Hb and other laboratory parameters were evaluated using Spearman's rank correlation coefficients. Correlations are presented as Spearman’s rho with corresponding two-sided p-values. Receiver operating characteristic (ROC) curve analysis was performed to evaluate the performance of RET-Hb in identifying iron deficiency anemia and to compare its diagnostic performance with that of serum ferritin, TSAT, serum iron, MCV, and MCH. The optimal cut-off value was determined using the maximum Youden index, and sensitivity, specificity, positive predictive value (PPV), negative predictive value (NPV), and their 95% confidence intervals (CIs) were calculated for the selected threshold. All tests were two-tailed, and a p-value < 0.05 was considered statistically significant.

## Results

A total of 200 participants were enrolled, including 100 cases diagnosed with IDA based on CBC reports and iron profile results, and 100 participants with normal hemograms and iron profiles who were included as the control group. The study included 119 females (59.5%) and 81 males (40.5%). Among the IDA group, 36 participants were male and 64 were female. The mean age in the IDA group was 57.64 ± 11.35 years. No statistically significant differences were observed between the case and control groups with respect to age or gender distribution. Compared with controls, individuals in the IDA group showed significantly lower mean values of serum ferritin, serum iron, TSAT, MCV, and MCHC (p < 0.05). Conversely, mean TIBC and RDW-CV were significantly elevated among patients with IDA. RET-Hb levels were substantially lower in the IDA group compared with the control group (Table 1).

**Table 1:** Baseline characteristics of study participants ^**^P < 0.001 is highly significant IDA: iron deficiency anemia; SD: standard deviation; TIBC: total iron-binding capacity; TSAT: transferrin saturation; MCV: mean corpuscular volume; MCH: mean corpuscular hemoglobin; MCHC: mean corpuscular hemoglobin concentration; RDW-CV: red cell distribution width-coefficient of variation; RET-Hb: reticulocyte hemoglobin

Variables	IDA group (n = 100)	Control group (n = 100)	P-value
				Age, years, mean ± SD	57.64 ± 11.35	55.96 ± 11.01	0.29
Gender, n (%)			
Male	36 (36%)	45 (45%)	0.195
Female	64 (64%)	55 (55%)	
Ferritin, ng/mL, mean ± SD	13.58 ± 6.04	54.59 ± 8.22	< 0.001^*^
Iron, mcg/dL, mean ± SD	24.64 ± 8.32	71.85 ± 12.47	< 0.001^*^
TIBC, mcg/dL, mean ± SD	487.15 ± 52.17	341.32 ± 22.28	< 0.001^*^
TSAT, %, mean ± SD	12.89 ± 5.33	31.49 ± 3.57	< 0.001^*^
Hemoglobin, g/dL, mean ± SD	7.46 ± 0.46	13.09 ± 0.79	< 0.001^*^
MCV, fL, mean ± SD	63.33 ± 5	84.36 ± 3.19	< 0.001^*^
MCH, pg, mean ± SD	20.49 ± 2.64	28.14 ± 1.17	< 0.001^*^
MCHC, g/dL, mean ± SD	29.43 ± 2.66	33.42 ± 0.95	< 0.001^*^
RDW-CV, %, mean ± SD	20.5 ± 3.55	13.92 ± 1.16	< 0.001^*^
RET-Hb, pg, mean ± SD	26.68 ± 4.04	32.68 ± 1.94	< 0.001^*^

**Figure 1:**
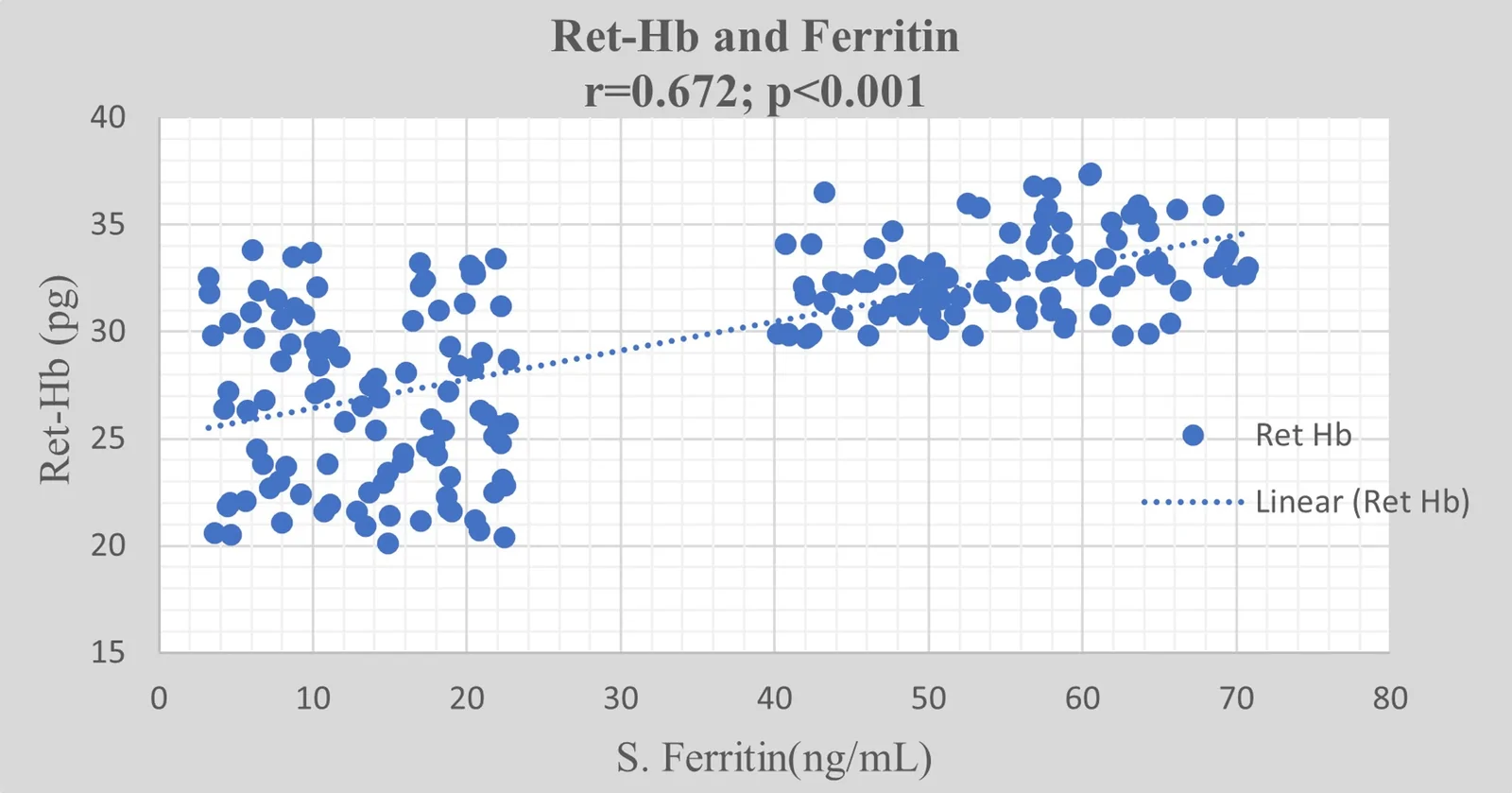
The association between RET-Hb and serum ferritin RET-Hb: reticulocyte hemoglobin

**Figure 2:**
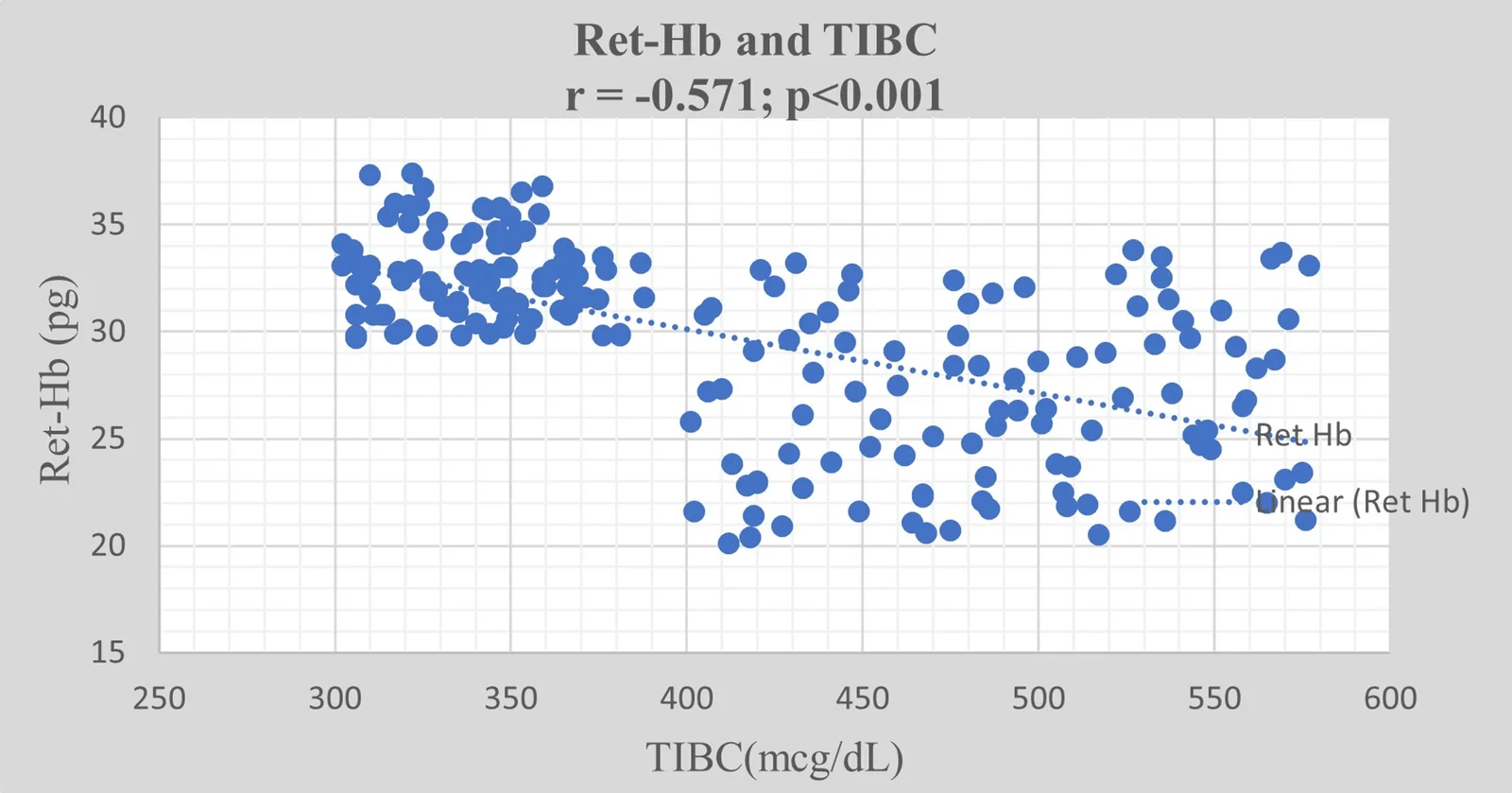
The association between RET-Hb and TIBC RET-Hb: reticulocyte hemoglobin, TIBC: total iron-binding capacity

**Figure 3:**
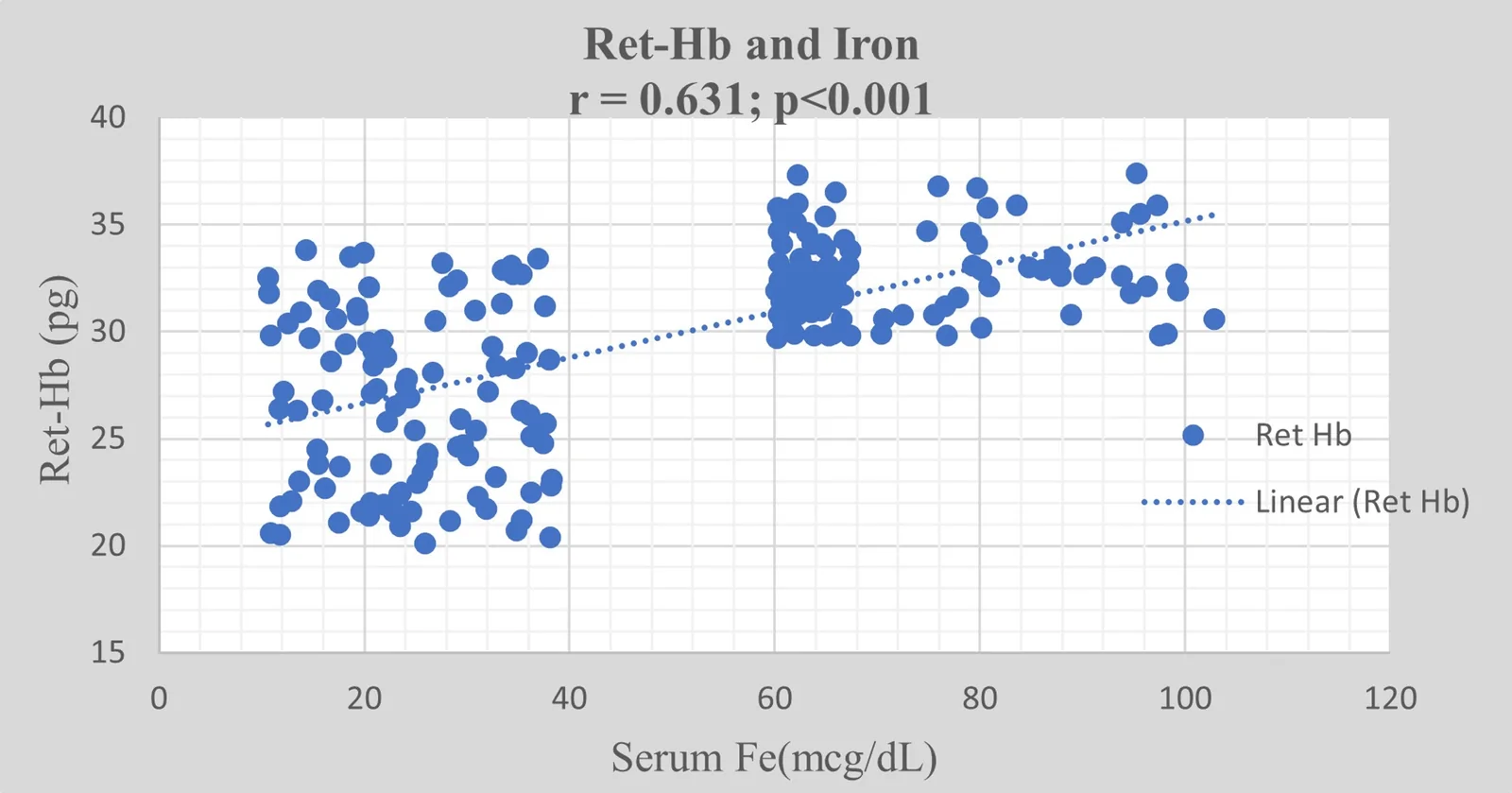
The association between RET-Hb and serum iron RET-Hb: reticulocyte hemoglobin

**Figure 4:**
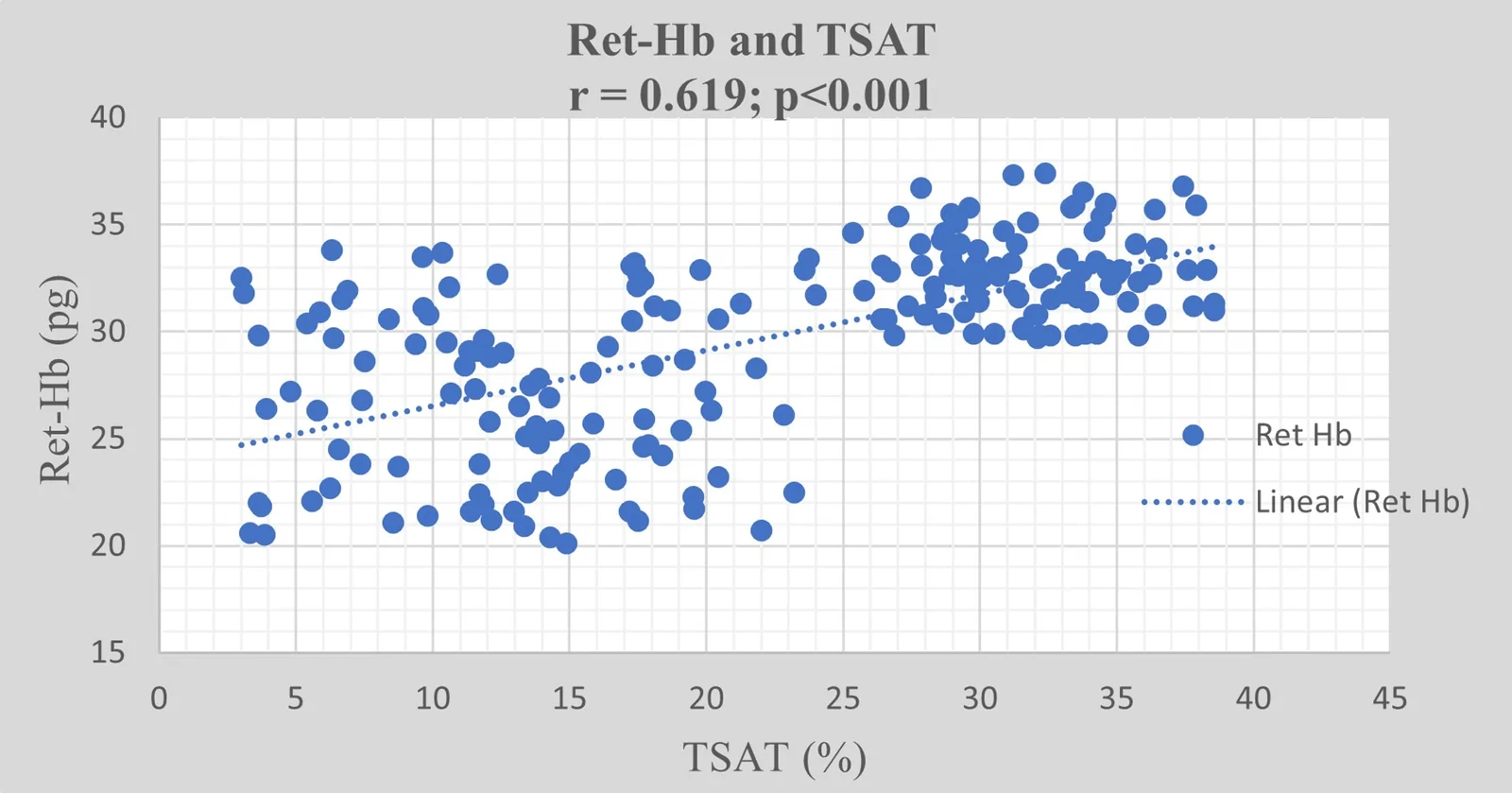
The association between RET-Hb and TSAT RET-Hb: reticulocyte hemoglobin; TSAT: transferrin saturation

Within the IDA cohort, baseline RET-Hb values showed strong positive associations with baseline serum ferritin levels (r = 0.672; p < 0.001), serum iron levels (r = 0.631; p < 0.001), and TSAT levels (r = 0.619; p < 0.001). Conversely, RET-Hb was negatively correlated with baseline TIBC levels (r = -0.571; p < 0.001). These correlations are illustrated in Figures 1-4.

**Figure 5:**
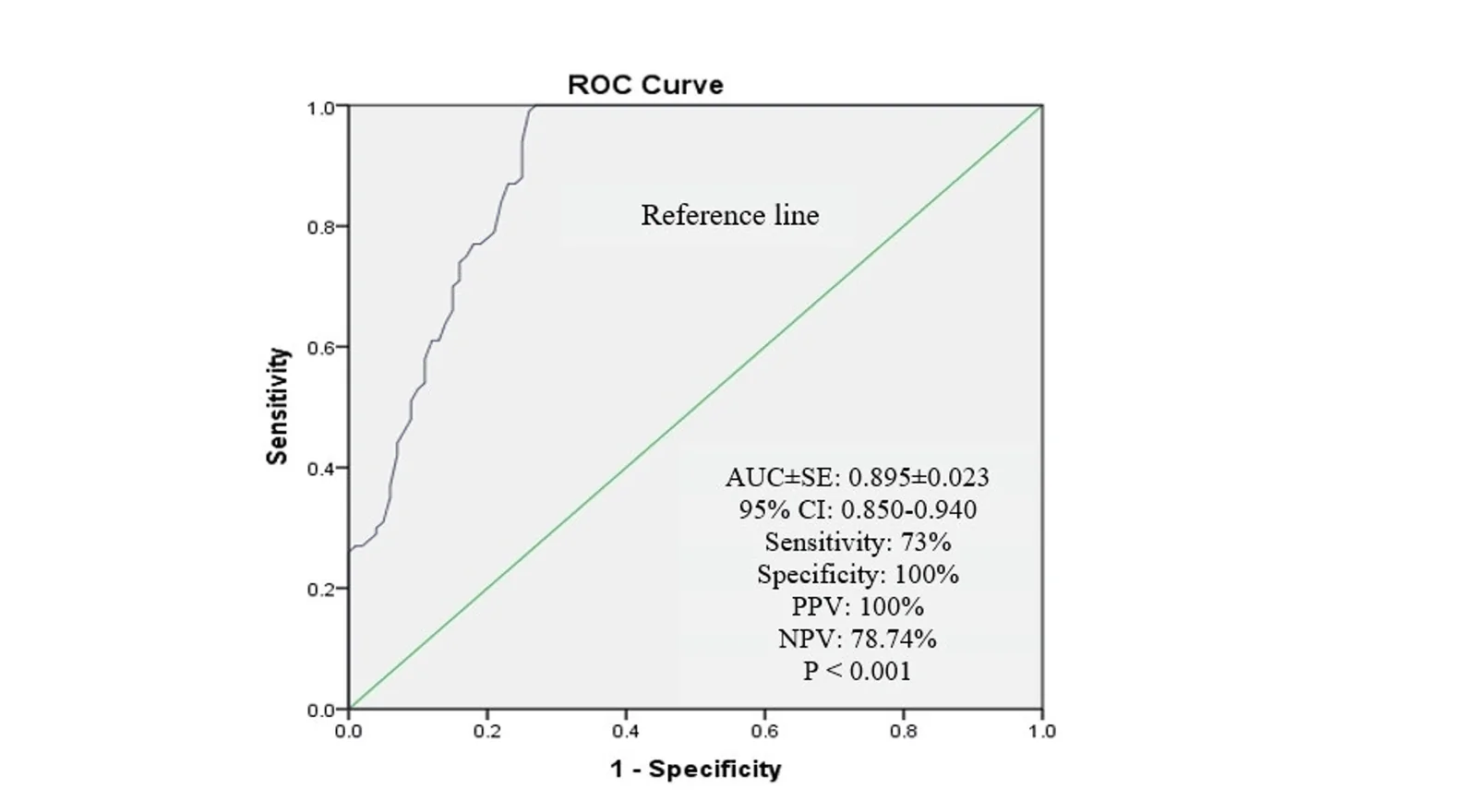
Efficacy of RET-Hb in the diagnosis of IDA RET-Hb: reticulocyte hemoglobin; IDA: iron deficiency anemia; ROC: receiver operating characteristic; PPV: positive predictive value; NPV: negative predictive value; CI: confidence interval; AUC: area under the ROC curve; SE: standard error

Figure 5 depicts the performance assessment of RET-Hb. ROC curve analysis was performed to evaluate the discriminatory ability of RET-Hb. RET-Hb demonstrated good discrimination between patients with IDA and healthy controls, with an AUC of 0.895 (95% CI: 0.850-0.940). The optimal RET-Hb threshold was selected using the maximum Youden index, calculated as sensitivity plus specificity minus one. Because lower RET-Hb values indicated iron deficiency anemia, a test result was considered positive when the value was less than or equal to the selected threshold. A RET-Hb threshold of 29.6 pg provided a sensitivity of 73.0% and a specificity of 100%, with a PPV of 100% and an NPV of 78.74% (p < 0.001) (Table 2).

**Table 2:** RET-Hb test characteristics based on optimal cutoff values determined by ROC curve analysis ET-Hb: reticulocyte hemoglobin; ROC: receiver operating characteristic

Characteristics	Value
Sensitivity	73.00%
Specificity	100.00%
Positive predictive value	100.00%
Negative predictive value	78.74%
Accuracy	86.50%

Where more than one threshold produced the same maximum Youden index, the threshold with higher specificity was selected (Appendix). The diagnostic performance of RET-Hb was compared with that of conventional iron-related parameters, including serum ferritin, TSAT, serum iron, MCV, and MCH. Serum ferritin and serum iron each achieved an AUC of 1.000. The corresponding AUCs for TSAT, MCV, and MCH were 0.999, 0.999, and 0.996, respectively. Pairwise comparison using DeLong’s test showed that the AUCs of the conventional indices were significantly higher than that of RET-Hb, with all comparisons yielding p-values below 0.001 (Appendix). Ordinary logistic regression showed separation because of the marked distinction between confirmed IDA cases and healthy controls.

**Figure 6:**
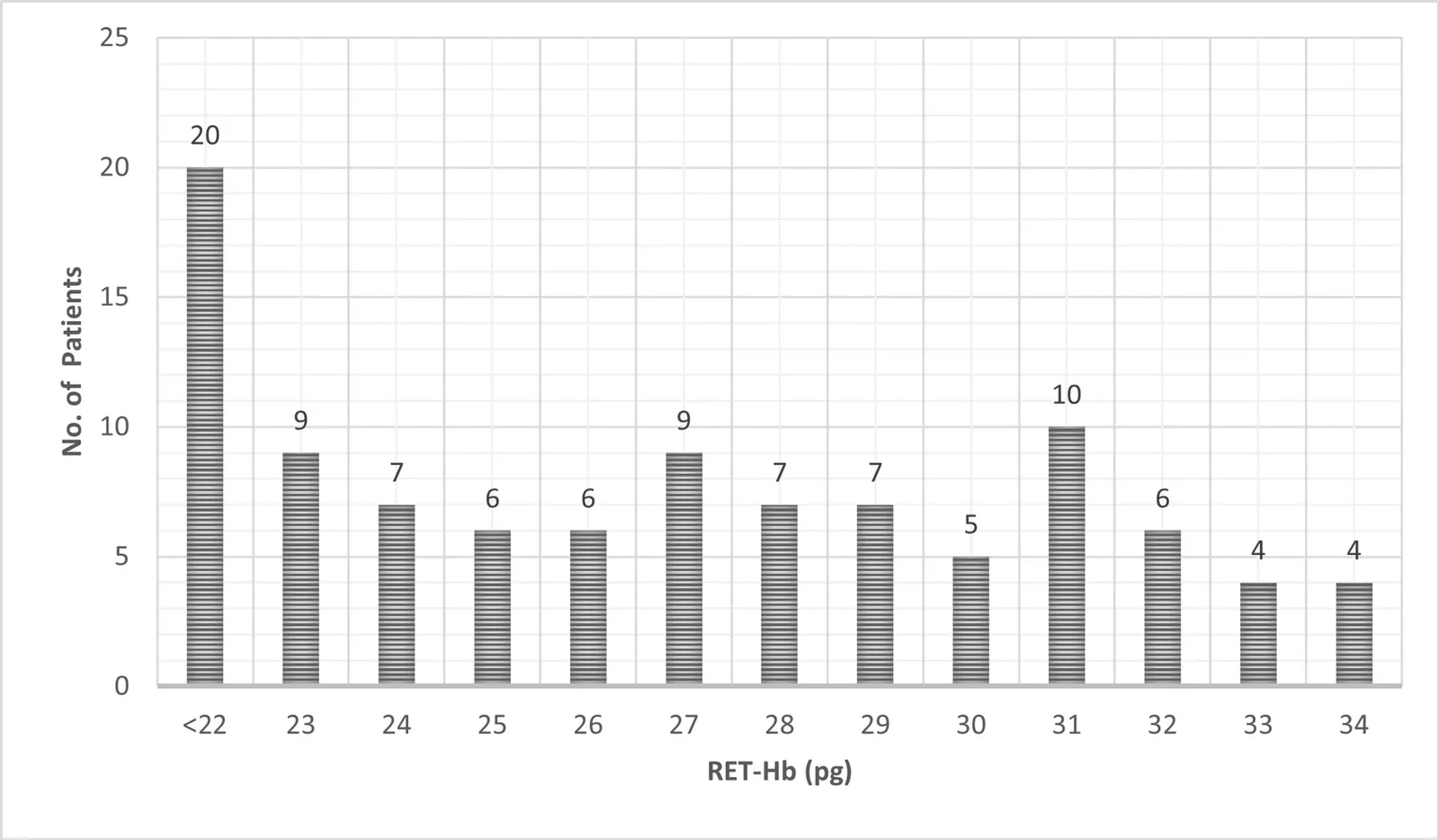
Distribution of RET-Hb in the IDA group RET-Hb: reticulocyte hemoglobin; IDA: iron deficiency anemia

The average RET-Hb level in patients with iron deficiency was 26.68 ± 4.04 pg, with a range of 20.4 to 33.8 pg, whereas the control group had an average RET-Hb level of 32.68 ± 1.94 pg, with a range of 29.7 to 37.4 pg. Among the 100 patients with iron deficiency in our study, 73 individuals had a RET-Hb level below 29.65 pg, while 27 had values of 29.65 pg or higher. This resulted in an overall sensitivity of 73.00% for detecting iron deficiency using this cut-off. In contrast, all participants in the control group, consisting of individuals with a normal CBC and iron profile, had RET-Hb values above 29.65 pg, yielding a specificity of 100.0%. Figure 6 depicts the distribution of RET-Hb levels in the IDA group. Tables 3-4 show the distribution of changes in RET-Hb levels from baseline after receiving standard intravenous (IV) iron therapy and their association with Hb responses, respectively. RET-Hb did not show a statistically significant pretreatment correlation with serum ferritin (Spearman's ρ = −0.061, p = 0.547), transferrin saturation (ρ = −0.040, p = 0.693), serum iron (ρ = −0.058, p = 0.567), MCV (ρ = −0.081, p = 0.425), hemoglobin (ρ = −0.134, p = 0.184), TIBC (ρ = 0.138, p = 0.172), or RDW-CV (ρ = 0.051, p = 0.613).

**Table 3:** Distribution of changes in RET-Hb (∆RET-Hb) levels pre- and post-iron infusion RET-Hb: reticulocyte hemoglobin

Category of Change in RET-Hb level (∆RET-Hb), pg	Number of patients
No change	0
< 1	20
1-2	35
2-3	16
3-4	9
4-5	6
> 5	14

**Table 4:** Association between ∆RET-Hb and Hb response after intravenous iron therapy RET-Hb: reticulocyte hemoglobin

Category of change in RET-Hb level (∆RET-Hb), pg	Mean Hb change in corresponding ∆RET-Hb category	Number of patients with Hb rise	Proportion of patients with Hb rise, %, n/N ×100
No change	0	0	0
< 1	0.035	5	25%
1-2	0.022	6	17%
2-3	0.018	4	25%
3-4	0.022	2	22%
4-5	0	0	0
> 5	0.035	5	36%

## Discussion

Anemia is a significant cause of morbidity in developing countries, leading to reduced work capacity and increased healthcare costs. Iron deficiency is associated with numerous adverse effects, including restless legs syndrome (RLS), diminished quality of life, fatigue, pica (especially pagophagia), impaired neurocognitive function, growth retardation, compromised immune function, an increased risk of preterm birth, adverse effects on cognitive performance, cardiovascular compromise, and infertility. Many of these conditions can be reversed with iron therapy. In chronic heart failure (CHF), iron deficiency is linked to an increased risk of mortality, irrespective of Hb levels. Therefore, the accurate and timely diagnosis of iron deficiency is crucial for preventing these adverse outcomes.

While a low ferritin level is highly specific for iron deficiency, its diagnostic utility can be limited in the presence of inflammation, where ferritin levels may be falsely elevated. Bone marrow aspiration remains the reference standard for diagnosis, requiring Prussian blue staining of bone marrow macrophages and erythroid precursors. However, it is not suitable for routine use because of its invasiveness and high cost. Various noninvasive biochemical parameters are also used to diagnose IDA, but their interpretation is complicated by factors that affect their accuracy. For instance, serum iron levels, serum ferritin levels, TSAT, and TIBC are the most widely used tests. However, the accuracy and diagnostic performance of these markers may be compromised under certain clinical conditions, such as inflammation or chronic disease, as previously discussed [2,3,6,7,8].

In this study, we investigated the potential of RET-Hb as an adjunctive biomarker for diagnosing and assessing early functional response in IDA. While conventional iron parameters - such as serum ferritin, serum iron level, TIBC, and TSAT - remain essential for diagnosing IDA, RET-Hb may serve as a reliable adjunct to enhance diagnostic accuracy. Moreover, due to its ability to reflect real-time iron availability, RET-Hb offers additional clinical value in assessing early functional response to iron therapy. Using a threshold of 29.65 pg/cell, RET-Hb demonstrated a PPV of 100% for ruling in iron deficiency. RET-Hb values can be readily obtained through a CBC using a multichannel hematological autoanalyzer, offering a convenient and accessible parameter for clinical assessment.

Our findings showed that patients with IDA exhibited markedly reduced RET-Hb values compared to healthy individuals of both sexes. Notably, RET-Hb thresholds may vary across different demographic and regional populations. Unlike traditional iron parameters, RET-Hb is generally considered less susceptible to acute-phase reactions, suggesting its utility as a consistent and clinically useful marker across various settings. This supports the utility of low RET-Hb as an adjunctive parameter for identifying IDA when interpreted alongside conventional iron indices, consistent with previously published data. In addition to RET-Hb, Hb, RBC indices, serum iron, TSAT, ferritin, TIBC, and RDW-CV were all significantly associated with IDA. Furthermore, RET-Hb demonstrated a strong correlation with Hb and iron profile parameters, reinforcing its potential role in the comprehensive evaluation of iron status.

Results from the comparison of the diagnostic performance of RET-Hb with conventional iron-related parameters, including serum ferritin, TSAT, serum iron, MCV, and MCH, require cautious interpretation because ferritin, serum iron, and transferrin saturation contributed to the diagnostic definition of IDA. Their near-perfect diagnostic performance therefore partly reflects the incorporation of these measurements into the reference standard rather than an entirely independent comparison with RET-Hb. In addition, the study compared patients with established IDA with healthy controls. This produced marked separation between the groups, particularly for MCV and MCH, and may have overestimated the diagnostic performance of conventional indices compared with what would be observed in an unselected clinical population. RET-Hb nevertheless retained good overall discrimination and provided complete specificity at the identified threshold. Its principal clinical value may lie in its rapid availability as part of an automated blood count, its ability to reflect iron availability to recently produced erythrocytes, and its potential role in early treatment monitoring.

The present results support RET-Hb as a useful complementary marker but do not establish its superiority over conventional indices for the initial diagnosis of established IDA. IDA was identified using a composite reference standard incorporating hemoglobin and conventional iron indices, including serum ferritin, serum iron, transferrin saturation, and TIBC. Because some of these indices were subsequently included in the comparative ROC analysis, their diagnostic performance may have been overestimated because of incorporation bias. The findings should therefore be interpreted as an exploratory comparison within a case-control population rather than an independent head-to-head validation against a definitive gold standard.

A RET-Hb change of < 1 pg was associated with only a very small mean Hb increase (0.035 g/dL), and only 25% of patients in this group showed a rise in Hb. Across the intermediate RET-Hb change groups (1-5 pg), the mean Hb increase remained minimal (0-0.022 g/dL), without a clear stepwise trend. Among patients with a ΔRET-Hb of > 5 pg, 36% showed a rise in Hb levels. Overall, these differences were small and inconsistent, and no statistically significant correlation was evident. Because Hb synthesis and release into the circulation typically require several days, any correlation with early changes in RET-Hb may not be apparent within 48 hours. RET-Hb increased significantly within 48 hours after intravenous iron, suggesting early improvement in functional iron availability that preceded measurable changes in hemoglobin.

All patients demonstrated some degree of improvement, as no individuals exhibited a lack of change in RET-Hb from baseline. A RET-Hb level change of < 1 pg was observed in 20 patients (20%), indicating a minimal response to iron therapy. A modest but potentially meaningful improvement was noted in 35 patients (35%). A change of 2-3 pg in RET-Hb was observed in 16 patients (16%), suggesting a moderate response to iron replenishment. Further stratification showed that RET-Hb changes of 3-4 pg, 4-5 pg, and > 5 pg were observed in nine (9%), six (6%), and 14 patients (14%), respectively. The data suggest that most patients experienced an increase in RET-Hb following iron therapy immediately after 48 hours. Our result on iron responsiveness using RET-Hb has been supported by several studies [9]. RET-Hb can be considered a useful marker for the diagnosis of IDA and as a potential indicator of early functional response following intravenous iron therapy.

In our study, an optimal RET-Hb threshold of 29.65 pg was identified, which demonstrated 73% sensitivity and 100% specificity. Values below this cut-off can effectively predict IDA. Uçar et al. reported that a RET-Hb level below 90% was associated with a sensitivity of 49.1% in their investigation [10]. In a similar vein, Toki et al. proposed a RET-Hb threshold of 28.5 pg, which demonstrated a sensitivity of 68% and a specificity exceeding 90% [11]. Conversely, Aedh et al. determined that a lower cut-off value of 21.2 pg per cell achieved 100% sensitivity, though specificity was 64.1% [12]. Furthermore, Sarin et al. identified a RET-Hb cut-off value of 30.4 pg among cancer patients, reporting corresponding sensitivity and specificity rates of 67.4% and 64.5%, respectively [13]. The variations in cut-off values may be attributed to differences in the degree of sensitivity used in each study.

This study has certain limitations that should be acknowledged while interpreting the findings. First, the study was conducted at a single tertiary care center, which may reflect the patient population and clinical practices specific to that setting. Second, the follow-up period after intravenous iron therapy was limited to 48 hours, which was designed to evaluate early functional response following intravenous iron therapy rather than long-term treatment outcomes. Although RET-Hb increased significantly within 48 hours following intravenous iron infusion, this short observation period reflects an early biological response and does not permit assessment of sustained hematological recovery or long-term clinical outcomes. Another important limitation of this study is that the control group consisted of healthy individuals rather than patients with alternative causes of anemia such as anemia of chronic inflammation, chronic kidney disease, thalassemia trait, or mixed nutritional deficiencies.

Consequently, the observed diagnostic performance, particularly the high specificity, may represent an optimal estimate and may not fully reflect performance in routine clinical practice where distinguishing IDA from competing etiologies is more challenging. (spectrum bias, inflated specificity) RET-Hb is an analyzer-specific parameter, and absolute values and optimal diagnostic thresholds may differ across hematology platforms. Therefore, the cut-off identified in this study should be externally validated before being applied to other laboratory systems. Diagnostic performance may therefore differ in routine clinical practice where alternative etiologies frequently coexist. Several studies have reported the effectiveness of RET-Hb in monitoring IV Iron therapy in patients with end-stage renal disease receiving long-term dialysis therapy. Additionally, some studies have highlighted the utility of RET-Hb in evaluating hematologic improvement following oral iron replacement therapy in patients affected by chronic inflammatory disorders, such as rheumatoid arthritis [14,15].

This study evaluated both the diagnostic performance of RET-Hb and its early change following intravenous iron therapy within a well-defined cohort of patients with IDA. The balanced inclusion of age- and sex-matched controls, assessment of conventional iron indices alongside RET-Hb, and use of an automated hematology analyzer enhance the clinical applicability of the findings. External validation in larger multicenter studies involving patients with diverse causes of anemia is required before widespread clinical implementation.

## Conclusions

Our study demonstrates that a RET-Hb threshold of 29.65 pg may serve as a supportive diagnostic indicator for IDA when interpreted together with standard iron indices such as serum ferritin, TSAT, and TIBC. Given its ability to reflect real-time iron availability, RET-Hb may also aid in monitoring the early therapeutic response, including changes detectable as early as 48 hours post-therapy, thereby contributing to more informed clinical decisions. This may help optimize iron therapy, potentially avoiding unnecessary supplementation, minimizing adverse effects, and improving resource utilization. RET-Hb demonstrated good diagnostic performance for identifying IDA and may serve as a useful adjunctive marker alongside conventional iron studies. In addition, RET-Hb increased significantly within 48 hours following intravenous iron infusion, suggesting early improvement in functional iron availability before measurable changes in Hb become apparent. However, because follow-up was limited to 48 hours, these findings should be interpreted as evidence of an early biological response rather than definitive treatment monitoring. The diagnostic performance observed in this study may have been influenced by the comparison of confirmed IDA cases with healthy controls rather than with patients having other causes of anemia. Larger prospective studies with longer follow-up are needed to establish the role of RET-Hb in longitudinal monitoring of iron therapy. Future studies should aim to include a broader range of anemia etiologies, such as pregnancy-induced anemia and chemotherapy-induced anemia, to better establish the diagnostic performance and specificity of RET-Hb in differentiating IDA from other forms of anemia.

## Appendices

(A) Data collection proforma/questionnaire

Study Title: Diagnostic Utility of Reticulocyte Hemoglobin in Iron Deficiency Anemia and Early Response Following Intravenous Iron Therapy

A structured data collection proforma was used to systematically record the demographic and laboratory parameters of all participants. For cases, baseline data were collected, and follow-up data were recorded at 48 hours for patients who had received intravenous iron therapy as part of standard care.

Section A*:* Participant Identification

Participant ID: __________

Age (years): __________

Sex: ☐Male

☐ Female

*Section B:* Baseline Hematological Parameters

Hemoglobin (g/dL): __________

Mean Corpuscular Volume (MCV) (fL): __________

Mean Corpuscular Hemoglobin (MCH) (pg): __________

Mean Corpuscular Hemoglobin Concentration (MCHC)(g/dL): __________

Red Cell Distribution Width (RDW-CV) (%): __________

Reticulocyte Hemoglobin (RET-Hb) (pg): __________

Section C: Baseline Iron Profile Parameters and C-Reactive Protein (CRP)

Serum Iron (mcg/dL): __________

Total Iron Binding Capacity (TIBC) (mcg/dL): __________

Serum Ferritin (ng/mL): __________

Transferrin Saturation (TSAT) (%): __________

C-Reactive Protein (CRP) (mg/L): __________

Section D:Treatment Details

Intravenous Iron Therapy Given as standard therapy ☐ Yes ☐ No

Type of Iron Preparation ☐ Ferric Carboxy-maltose

Dose administered: __________

Section E:Hematological Parameters, 48 hr after receiving standard treatment [Cases Only]

Hemoglobin (Hb post-treatment) (g/dL): __________

Change in Hemoglobin (ΔHb): __________

RET-Hb (post-treatment) (pg): __________

Change in RET-Hb level (ΔRET-Hb) (in pg): __________

**Table 5:** Category of change in RET-Hb level (ΔRET-Hb) (in pg) for patients RET-Hb: reticulocyte hemoglobin

Category	Tick (✓)
No change (0 pg)	☐
< 1 pg	☐
1-2 pg	☐
2-3 pg	☐
3-4 pg	☐
4-5 pg	☐
> 5 pg	☐

(B) Details of sample size calculation

The sample size was estimated using a precision-based approach for a case-control diagnostic accuracy study. Based on previous studies, an expected sensitivity of 85% and specificity of 90% for RET-Hb were assumed. Using a two-sided 95% confidence level and an absolute precision of 7%, the minimum required sample size was 100 patients with iron deficiency anemia and 71 controls. To improve the precision of specificity estimation and maintain a balanced case-control design, 100 age- and sex-matched controls were included. Using the precision-based formula for a case-control diagnostic accuracy study, sample size was calculated.

The expected sensitivity of 85% was selected based on the overall body of published evidence evaluating the diagnostic performance of reticulocyte hemoglobin (RET-Hb) for iron deficiency. Previous studies have reported sensitivities ranging from 49.1% to 100%, with substantial variability attributable to differences in study populations, disease spectrum, analyzer platforms, diagnostic cut-off values, and reference standards. Rather than adopting the lowest or highest reported estimate, an expected sensitivity of 85% was considered a clinically reasonable and representative value for sample size estimation.

(C) ROC curve analysis for RET-Hb and its comparison with conventional iron parameters

The optimal RET-Hb threshold was selected using the maximum Youden index, calculated as sensitivity plus specificity minus one. Because lower RET-Hb values indicated iron deficiency anemia, a test result was considered positive when the value was at or below the selected threshold. Where more than one threshold produced the same maximum Youden index, the threshold providing the higher specificity was selected.

The optimal threshold of ≤29.65 pg was selected using the maximum Youden index. The maximum Youden index for RET-Hb was 0.73. Two adjacent thresholds produced the same maximum index. A cut-off of ≤29.65 pg was selected because it provided a sensitivity of 73.0% and a specificity of 100%, whereas a threshold of ≤29.75 pg provided a sensitivity of 74.0% and a specificity of 99.0%. The more specific threshold was retained for reporting.

**Table 6:** Comparison between two cutoff values with the same Youden index

Cutoff	Sensitivity	Specificity	Youden index
≤ 29.65 pg	73%	100%	0.73
≤ 29.75 pg	74%	99%	0.73

The diagnostic performance of RET-Hb was compared with that of conventional iron-related parameters, including serum ferritin, transferrin saturation (TSAT), serum iron, mean corpuscular volume (MCV), and mean corpuscular hemoglobin (MCH). Serum ferritin and serum iron each achieved an AUC of 1.000. The corresponding AUCs for transferrin saturation, MCV, and MCH were 0.999, 0.999, and 0.996, respectively. Pairwise comparison using DeLong’s test showed that the AUCs of the conventional indices were significantly higher than that of RET-Hb, with all comparisons yielding P values below 0.001.

**Table 7:** Comparison of the diagnostic performance between RET-Hb and iron-related parameters RET-Hb: reticulocyte hemoglobin; CI: confidence interval; TSAT: transferrin saturation; MCV: mean corpuscular volume; MCH: mean corpuscular hemoglobin

Parameter	AUC (95% CI)	Optimal cutoff	Sensitivity	Specificity	P-value versus RET-Hb
RET-Hb	0.895 (0.850–0.940)	29.60 pg	73%	100%	Reference
Ferritin	1.000 (1.000–1.000)	22.72 ng/mL	100%	100%	< 0.001
TSAT	0.999 (0.998–1.000)	23.22%	99%	99%	< 0.001
Serum iron	1.000 (1.000–1.000)	38.31 µg/dL	100%	100%	< 0.001
MCV	0.999 (0.996–1.000)	72.30 fL	99%	100%	< 0.001
MCH	0.996 (0.991–1.000)	24.80 pg	100%	98%	< 0.001

The distribution of continuous variables was assessed using the Shapiro-Wilk test and visual inspection of histograms. Because RET-Hb and most conventional hematological and iron indices were not normally distributed, associations between RET-Hb and the other laboratory parameters were evaluated using Spearman rank correlation coefficients. Correlations are presented as Spearman’s rho with corresponding two-sided P values
